# Tissue and Salivary NMR Metabolomics in Reticular‐Type Oral Lichen Planus

**DOI:** 10.1002/nbm.70137

**Published:** 2025-09-03

**Authors:** Giacomo Setti, Anna Gambini, Valeria Righi, Adele Mucci, Thelma A. Pertinhez, Elena Ferrari, Mariana Gallo, Brunella Biscussi, Rita Antonelli, Marco Meleti, Cristina Magnoni

**Affiliations:** ^1^ Dentistry and Oral‐Maxillofacial Surgery Unit University of Modena and Reggio Emilia Modena Italy; ^2^ Department of Chemical and Geological Sciences University of Modena and Reggio Emilia Modena Italy; ^3^ Department for Life Quality Studies University of Bologna Rimini Italy; ^4^ Laboratory of Biochemistry and Metabolomics, Department of Medicine and Surgery University of Parma Parma Italy; ^5^ Instituto de Química del Sur, Departamento de Química Universidad Nacional del Sur and Consejo Nacional de Investigaciones Científicas y Técnicas Bahía Blanca Argentina; ^6^ Centro Universitario Odontoiatria University of Parma Parma Italy; ^7^ Dermatology Unit, Department of Surgical, Medical, Dental & Morphological Sciences With Interest Transplant, Oncological & Regenerative Medicine University of Modena and Reggio Emilia Modena Italy

**Keywords:** metabolomics, nuclear magnetic resonance, oral lichen planus, oxidative stress, saliva, tissue

## Abstract

Oral lichen planus (OLP) is a chronic T‐cell–mediated autoimmune disease, with low potential for malignant transformation. Its etiology remains unclear, necessitating immunohistochemical and molecular‐level studies to enhance diagnosis and management. Thirteen patients diagnosed with OLP and 13 healthy controls (HCs) were enrolled from three centers. Mucosal tissue samples collected during diagnostic biopsies and unstimulated whole saliva samples were analysed. A comprehensive approach was taken, with high‐resolution magic angle spinning (HR‐MAS) ^1^H‐NMR spectroscopy performed on biopsies and liquid ^1^H‐NMR spectroscopy on saliva samples to identify potential biomarkers correlated with OLP. Multivariate analyses effectively distinguish OLP patients from HC based on metabolic profiles, with key metabolites contributing to the separation. In tissue, triglycerides were significantly elevated in OLP biopsies, whereas amino acids such as glutamate, glutamine, taurine, glycine and alanine were significantly decreased in OLP tissues compared with controls (*p* < 0.05). Salivary analysis revealed significant alterations in compounds of bacterial origin—such as isobutyrate, isocaproate, isovalerate and agmatine—suggesting dysbiosis in OLP patients. The metabolic alterations identified highlight the roles of oxidative stress and lipid metabolism in OLP pathogenesis and suggest potential biomarkers for OLP diagnosis. These findings provide new insights into the molecular mechanisms of OLP, which may have important clinical implications for future diagnostic and therapeutic strategies.

## Graphical abstract

Oral lichen planus (OLP) is an autoimmune disease with a low potential for malignant transformation, and its origin remains unclear. Analysis of mucosal tissue biopsies and saliva samples by NMR spectroscopy helps to identify potential biomarkers related to OLP. Multivariate analyses effectively distinguish OLP patients from healthy controls based on metabolic profiles. These metabolic profiles improve our understanding of OLP pathogenesis by highlighting the role of oxidative stress and lipid dysregulation and suggest potential biomarkers to improve diagnosis.

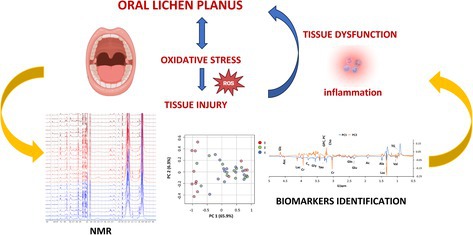

**Abbreviations**
3PhP: 3‐phenylpropionate
4OHPhA: 4‐hydroxyphenylacetate
Ac: acetate
Ade: adenine
Agm: agmatine
Asc: ascorbate
Cho: choline
Cr: creatine, Crn, creatinine
DMFT: decayed, missing, filled teeth index
EA: ethanolamine
FMPS: full mouth plaque score
For: formate
GPC: glycerophosphocholine
HR‐MAS: high‐resolution magic angle spinning
iBu: isobutyrate
iCa: isocaproate
iVa: isovalerate
Lac: lactate
MeOH: methanol
Oligo: a signal at 5.44 ppm, assigned to 1′‐CH and 1″‐CH of maltose/maltotriose
PC: phosphocholine
PCA: principal component analysis
PLS‐DA: partial least squares discriminant analysis
Pyr: pyroglutamate
Suc: succinate
Tau: taurine
TG: triglycerides
TSP: 3‐trimethylsilyl propanoic acid
Ur: uracil
VIP: variable importance projection

## 1 Introduction

Oral lichen planus (OLP) is a common T‐cell–mediated chronic autoimmune disease affecting mucocutaneous tissues; its etiology is not fully understood and involves multiple antigens and several precipitating factors such as drugs, stress, viral infections and dysbiosis [1, 2, 3]. OLP has a relatively high global incidence rate of 0.89%–2%, with a prevalence of around 1% worldwide; this disease predominantly affects females (2–1.4:1) and is more commonly observed in middle‐aged adults, with an increasing prevalence after the fourth decade of life [4, 5, 6]. Classified as a potentially malignant disorder, OLP has low reported transformation rates (0.28%–1.14%), but because of its clinical features, development site, disease evolution and reported symptoms, follow‐up and therapies are tailored accordingly [7, 8].

OLP pathogenesis involves the increased production of genetically induced polymorphic cytokines by T‐helper cells. This results in T‐cell activation and interaction with molecules involved in cell adhesion and signalling pathways, such as collagen, laminins and integrins. Tumour necrosis factor‐alpha (TNF‐α) and interleukins (IL‐1, ‐8, ‐10, ‐12) produced by basal membrane epithelial cells facilitate T‐cell chemotaxis, leading to immune cells binding to keratinocytes. The consequent upregulation of p53 and matrix metalloproteinase‐1 (MMP1) causes apoptosis and destruction of basal cells. Inflammation progresses towards chronicity, characterized by keratinocyte hyperproliferation, vacuolization of basal cells and a band‐like inflammatory infiltrate [1, 9].

The clinical oral appearance of OLP is highly variable and can be divided into several subtypes: reticular, papular, plaque, atrophic/erosive and ulcerative. The most common manifestation is a bilateral, keratotic reticular pattern involving the buccal mucosa, gingiva, lateral tongue and lower lip. Papular manifestations involve the lining mucosa or palate with small, white, raised lesions. The plaque subtype is common on the tongue dorsum and can be accompanied by white reticulation. Most of the patients presenting with these subtypes are asymptomatic. The atrophic/erosive and ulcerative patterns describe a more complex mucosal involvement with erythematous areas, extensive desquamation or distinct ulceration. Such lesions are always symptomatic, and symptoms are related to food intake or daily routine [4, 10].

As the clinical and histological appearance of the oral mucosa may not truly represent the damage that occurs at the genetic and epigenetic level in determining the onset and progression of the lesion, understanding the molecular mechanisms involved in OLP could be useful for developing new therapies. In this context, investigating the metabolite composition of saliva and/or oral tissues might also yield new insights into this pathology [11].

Metabolomics has emerged as a valuable tool for assessing metabolic changes related to health status and pathological alterations in biological systems. It assesses the levels of a wide range of endogenous and exogenous small molecules such as lipids, amino acids, peptides, nucleic acids, organic acids, vitamins, thiols and carbohydrates. Few studies report on the use of NMR spectroscopy to study salivary metabolic profiles and obtain a rapid overview of significant changes in OLP patients [12, 13, 14, 15, 16, 17, 18, 19, 20, 21]. However, the currently available data on the metabolic profiles of OLP patients compared with healthy controls (HCs) do not yet allow for the identification of the active metabolic pathways in this disease and the determination of their impact on its progression.

Tissue HR‐MAS NMR captures metabolic alterations occurring in situ at the lesional microenvironment, whereas saliva reflects the integrated output of salivary gland secretions, crevicular fluid and oral microbiota. Analysing both matrices, therefore, allows us to link local pathophysiologic features with whole‐mouth alterations. Furthermore, this information enhances our understanding of how local lesions affect saliva composition. Since saliva is a non‐invasive biofluid for sampling, these insights are particularly valuable as they could serve as a basis for developing future chairside diagnostic tools.

This study employed an NMR‐based ^1^H‐NMR and ^1^H high‐resolution magic angle spinning (HR‐MAS) NMR metabolomics approach to investigate the metabolic alterations associated with OLP, in saliva and oral mucosal tissue, respectively. To our knowledge, tissue analysis of OLP using HR‐MAS NMR spectroscopy has never been conducted, and only one study on the saliva of OLP subjects using NMR spectroscopy has been reported [16]. This study aimed to comprehensively characterize the metabolite profiles of oral mucosa biopsies and saliva samples to identify candidate biomarkers for OLP. Additionally, we evaluated the translational relevance of these findings to support future applications in preclinical modelling and clinical practice.

## 2 Experimental

### 2.1 Participants

The present study was approved by the Ethics Committee of the ‘Area Vasta Emilia Nord’ (AVEN) (protocol number: 38/2017/TESS/AUOMO‐509/2019/TESS/UNIPR). The patients and the healthy controls were enrolled in three centers, two based in Modena (Dermatology and Dentistry Units, Modena University Hospital) and one in Parma (University Dentistry Center of the University of Parma). According to the Declaration of Helsinki, written consent was obtained from all the volunteers who participated in this study. Strict selection criteria were applied to obtain the most homogeneous study population. Patients with a history of head and neck radiotherapy, HIV infection, lymphoma, sarcoidosis, autologous bone marrow transplantation and use of anticholinergic drugs were excluded. Specimen anonymization took place at the enrollment stage. The study cohort comprises 13 OLP patients (4 males, 9 females, 43–79 years) diagnosed by oral clinical and histological examination; to minimize biological heterogeneity across clinical variants, we prospectively limited inclusion to patients with bilateral reticular OLP. Erosive or ulcerative lesions, which carry a higher inflammatory burden and may show distinct metabolomic profiles, were excluded from this exploratory cohort. Thirteen healthy individuals (5 males, 8 females, 19–71 years) were enrolled in the control group. Mean number of decayed, missing, filled teeth index (DMFT) and full mouth plaque score (FMPS) were registered in all participants, during a comprehensive oral evaluation before specimen collection.

Further sample analysis was blindly conducted, and NMR operators were unaware of participant status.

### 2.2 NMR Tissue Analysis

#### 2.2.1 Tissue Sample Collection and Preparation for HR‐MAS Measurements

Samples from the OLP group were obtained during diagnostic biopsy procedures performed under local anesthesia. A distal nerve block was administered to minimize contamination from anesthetic drugs. An additional perilesional (P) mucosa sample was collected from clinically healthy tissue for each patient. For the healthy control group, mucosa samples were harvested during elective oral surgery procedures, such as tooth extractions or others. All specimens were divided into two parts: one underwent formalin fixation for routine histological analysis; the other was placed into a sterile tube, immediately flash‐frozen in liquid nitrogen, and then transferred to a −80°C freezer for storage until metabolomic analysis. To prevent thawing, tubes were transported using dry ice‐filled boxes to maintain the cold chain. Each tube was labeled with a unique sequential code assigned to each participant during anonymization procedures.

Each frozen biopsy sample was weighed (1–25 mg), introduced into a zirconia rotor (12‐ or 50‐μL capacity, depending on the sample amount) without any pretreatment, and 10 μL of D_2_O (99.9%) was added. The rotor was closed with a Teflon insert and a drive cap [22].

#### 2.2.2 NMR Data Collection and Analysis

One‐ and two‐dimensional NMR spectra were acquired with an NMR Bruker Avance III HD 600 MHz spectrometer (BrukerBiospin, Rheinstetten, Germany), equipped with a ^1^H, ^13^C, ^31^P HR‐MAS probe and a Bruker cooling unit, working at 600.13 MHz on ^1^H. All experiments were conducted at a 4 kHz spin rate and 5°C to slow tissue degradation. In detail, one‐dimensional water‐suppressed ^1^H HR‐MAS NMR (zgcppr sequence in Bruker library), water‐suppressed spin‐echo (Carr–Purcell–Meiboom–Gill [CPMG], cpmgpr sequence) and diffusion‐edited (ledbpgp2s1d sequence) experiments were conducted. Furthermore, two‐dimensional homonuclear ^1^H,^1^H COSY, TOCSY and ^1^H,^13^C heteronuclear single quantum coherence (HSQC) experiments were performed, as previously described [22]. The CPMG pulse sequence was used to highlight the narrow signals of metabolites and attenuate the broad signals of macromolecules and lipids. These were the spectra used for statistical analysis. ^1^H HR‐MAS CPMG NMR spectra were acquired using 4 s water presaturation, 2.27 s acquisition time, 109,132 data points, 40 ppm spectral width, a total echo time of 0.36 s (2‐ms single echo) and 512 scans. CPMG spectra were transformed with 1 Hz line broadening, manually phased, baseline corrected, aligned and binned (0.002 ppm) with MNova software package (MestReNova, ver. 11 Mestrelab Research S. L., Santiago de Compostela, Spain). Spectral regions that include signals of the exogenous anesthetic articaine (1.95–2.3 ppm, 2.6–3.0 ppm, 3.06–3.17 ppm, 3.8–3.9 ppm, 4.0–4.47 ppm, 7.45–7.6 ppm) were excluded from statistical analysis. The chemical shift was calibrated using the doublet of alanine set at 1.48 ppm. Each spectrum was normalized to the total area. The areas of selected signals from 26 out of 37 identified metabolites were estimated by deconvolution through the MNova Line Fitting routine on the 64k transformed normalized spectra. The identification of metabolites was based on literature data, on HMDB (http://www.hmdb.ca, version 5.0) and BMRB (https://bmrb.io) digital libraries and on our knowledge about the shape and position of the signals checked by two‐dimensional experiments (COSY, TOCSY and HSQC).

### 2.3 NMR Saliva Analysis

#### 2.3.1 Saliva Collection and Sample Preparation

The protocol for saliva collection was described previously [14]. For each participant, a sample of whole saliva was collected without stimulation. Immediately before collection, patients rinsed their mouths with water for 1 min. During collection (5–15 min), saliva samples were transferred to a tube containing NaN_3_ (0.5% final concentration) and kept on ice until a volume of approximately 2 mL was obtained and then frozen at −80°C. Each frozen saliva sample was thawed on ice and centrifuged at 15,000 × *g* for 10 min at 4°C to remove eukaryotic and prokaryotic cells, cellular debris and mucins, according to Quartieri et al. [23]. For NMR sample preparation, 1 mL of each saliva supernatant was ultra‐filtered using Amicon Ultra‐4 Centrifugal filters (3000 MWCO, Merck Millipore) at 4000 × *g* at 10°C for 60 min to deplete proteins that may interfere with metabolite quantification by NMR [24]. Then, ultra‐filtered supernatants were lyophilized and the dry residues were resuspended in 600 μL of 25 mM phosphate buffer pH 7.4, containing 1.45 mM 3‐trimethylsilyl propanoic acid (TSP), used as a quantitative standard, and 5% D_2_O for the solvent signal lock.

#### 2.3.2 ^1^H‐NMR Saliva Spectra Acquisition

One‐dimensional ^1^H‐NMR spectra of saliva samples were acquired at 25°C with a JEOL 600‐MHz ECZ600R spectrometer (JEOL Inc., Tokyo, Japan) as described in [25]. The spectra were processed, after zero‐filling to 256 K points, using a line broadening of 0.5 Hz.

### 2.4 Metabolomics Statistical Data Analysis

Univariate and multivariate statistical analyses were conducted using Metaboanalyst 6.0 (https://www.metaboanalyst.ca), a web‐based metabolomics data analysis software [26]. For tissue samples, multivariate statistical analysis was applied to the spectra after Pareto scaling. The area‐normalized data set was obtained by deconvolution and analysed after autoscaling. For saliva samples, the identification and quantitation of detected metabolites were performed using the Chenomx NMR Suite 9.0 software (Chenomx Inc., Edmonton, AB, Canada). Metabolite concentrations in each sample were normalized to the sum and then autoscaled across samples. Multivariate statistical analysis was performed using principal component analysis (PCA) and partial least squares discriminant analysis (PLS‐DA). Results were visualized as 2D score plots, and metabolites with high PLS‐DA variable importance projection (VIP) scores were identified. PLS‐DA cross‐validation was performed using five components and a fivefold CV method on the Metaboanalyst platform. Cross‐validation results are reported in Figures S2 and S3. False discovery rate (FDR) correction and Fisher's least significant difference method (Fisher's LSD) were performed after one‐way ANOVA. The univariate analysis on saliva data generated a volcano plot using fold change ≥ 1.5 and *p* ≤ 0.05 as thresholds.

## 3 Results

### 3.1 Clinical Features of OLP Cases

All 13 included cases presented with a bilateral reticular pattern. The lining mucosa of the cheeks was solely involved in seven cases. In the remaining six cases, one or more additional sites were involved, such as the tongue, lip and gingiva. The presence of other manifestations combined with Wickham's striae was detected in only five cases, of which four showed a papular pattern, and one exhibited a slightly erosive pattern.

Most OLP samples and all perilesional normal mucosa samples were harvested from the lining mucosa of the cheeks, with no side predilection. One OLP sample was taken from the gingiva. Smoking was reported in five and alcohol consumption in two cases. FMPS was 41% ± 35%. Caries prevalence was calculated using the DMFT, with a mean value of 18.8 ± 5.3.

### 3.2 ^1^H HR‐MAS NMR Tissue Analysis

All spectra obtained from the 26 tissue samples from OLP patients (lesion and perilesional biopsies) and the 13 controls from healthy subjects were included in the analyses. We identified 37 metabolites in the region between 0.70 and 9.0 ppm of each spectrum, excluding signals from the solvent and the anesthetic articaine. All assignments are listed in Table S1.

**FIGURE 1:**
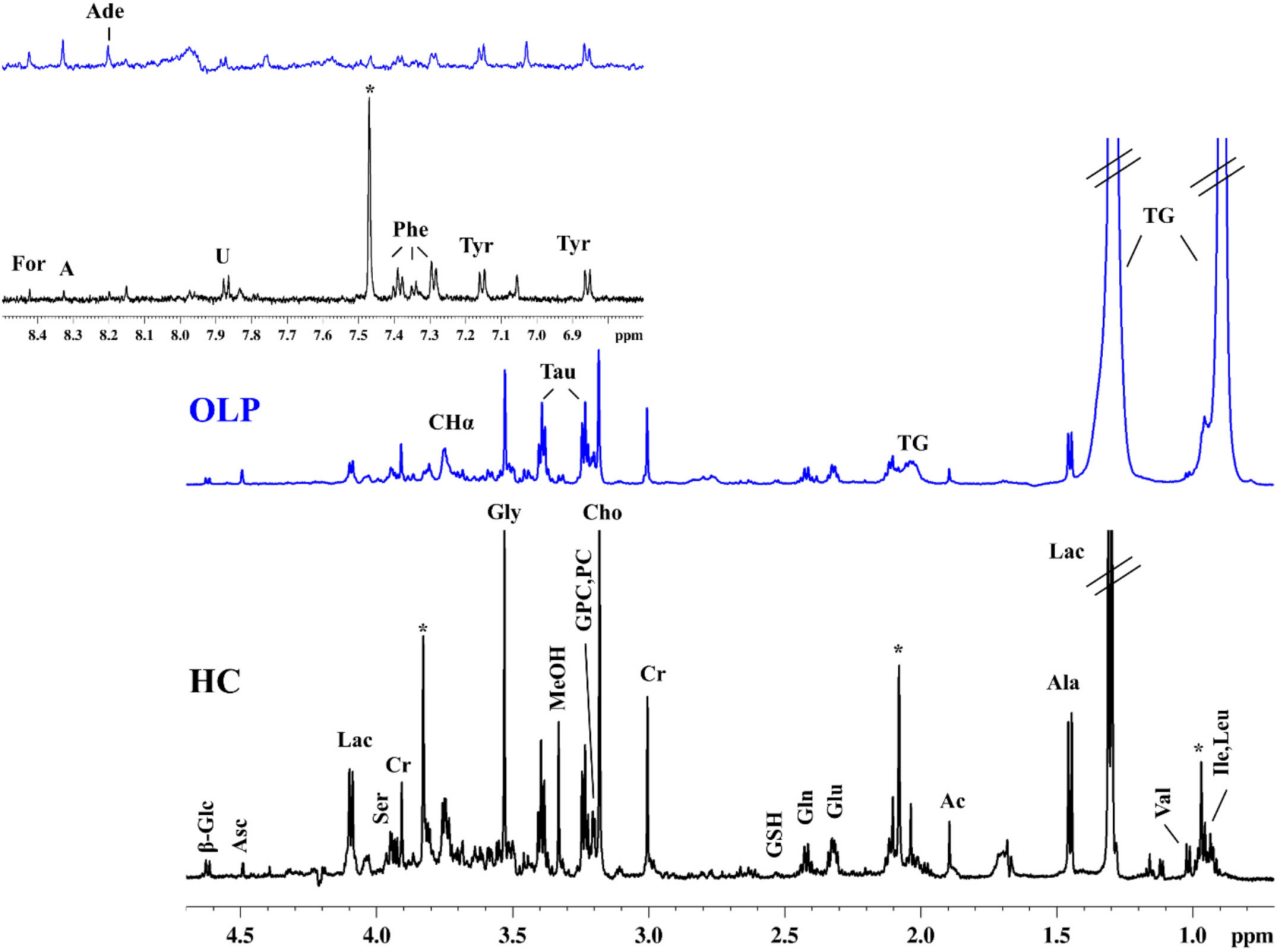
^1^H HR‐MAS CPMG NMR spectra from alveolar mucosa of a healthy control (HC) subject (black) and from buccal mucosa of an oral lichen planus (OLP) subject (blue). A: adenosine, Ac: acetate, Ade: adenine, Ala: alanine, Asc: ascorbate, Cho: choline, CHα: signals of CHα from alanine, Cr: creatine, For: formate, Glc: glucose, Gln: glutamine, Glu: glutamate, Gly: glycine, GPC: glycerophosphocholine, GSH, glutamine and glutamate, GSH: glutathione, Ile: isoleucine, Lac: lactate, Leu: leucine, MeOH: methanol, PC: phosphocholine, Phe: phenylalanine, Ser: serine, Tau: taurine, TG: triglyceride, Tyr: tyrosine, U: uridine, Val: valine. *Articaine signals (exogenous anaesthetic).

A representative ^1^H HR‐MAS CPMG NMR spectrum of OLP is reported in Figure 1, where the assignments of the major signals are indicated. The aliphatic region (0.7–4.8 ppm) is richer in metabolites compared to the aromatic one (6.9–9.0 ppm). The following metabolites were identified: isoleucine (Ile), leucine (Leu), valine (Val), triglycerides (TG), alanine (Ala), acetate (Ac), glutamate (Glu), glutamine (Gln), glutathione (GSH), succinate (Suc), creatine (Cr), choline (Cho), glycerophosphocholine (GPC), phosphocholine (PC), taurine (Tau), methanol (MeOH), glycine (Gly), lactate (Lac), ascorbate (Asc), β‐glucose (β‐Glc), tyrosine (Tyr), phenylalanine (Phe), adenine (Ade), adenosine (A) and formate (For). In addition, a broad peak corresponding to the overlapped signals of the hydrogen α (Hα) of diverse amino acids was observed in the spectra.

**FIGURE 2:**
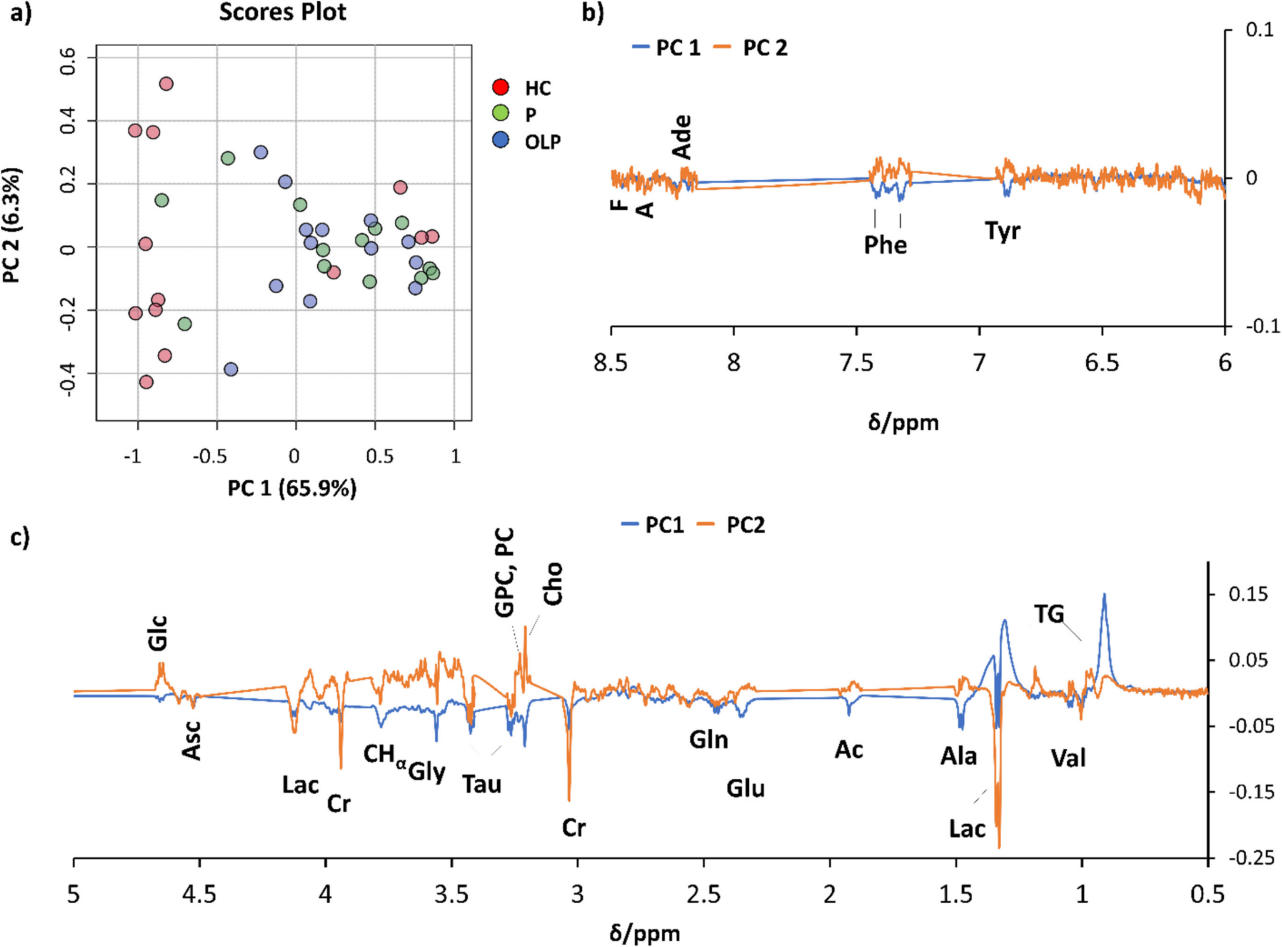
Principal component analysis (PCA) scores plot (a) and aromatic (b) and aliphatic (c) regions of PC loadings obtained from ^1^H HR‐MAS CPMG NMR spectra of HC (healthy control, red), P (perilesional, green) and OLP (oral lichen planus, blue) tissue samples. A: adenosine, Ac: acetate, Ade: adenine, Ala: alanine, Asc: ascorbate, Cho: choline, CHα: signals of CHα from alanine, glutamine and glutamate, Cr: creatine, For: formate, Glc: glucose, Gln: glutamine, Glu: glutamate, Gly: glycine, GPC: glycerophosphocholine, Lac: lactate, PC: phosphocholine, Phe: phenylalanine, Tau: taurine, TG: triglyceride, Tyr: tyrosine, Val: valine.

Exploratory multivariate analysis (Figure 2) was performed on the normalized spectra of the tissues and on the deconvoluted areas of selected peaks to highlight differences between the three sample classes: HC, P and OLP.

PCA performed on the normalized spectra does not show a clear separation among classes (Figure 2), even though the larger part of HC samples is found at negative PC1 < −0.7 and most P and OLP samples are found at PC1 > −0.5. Inspection of PC1 loadings shows that nine out of 13 HCs are characterized by a higher abundance of small metabolites and a lower abundance of TG with respect to P and OLP samples. A slight improvement in the clusterization of OLP samples is obtained when using PLS‐DA (Figure S1); HC samples are still separated into two groups, and P samples remain scattered. Inspection of the first latent variable (LV1) loading profile (Figures S1b and S1c) confirms that OLPs and Ps are characterized, on average, by a higher level of TG. In contrast, HCs are richer in the other metabolites, even though four HC samples (those with positive LV1) display non‐negligible TG signals. As for second latent variable (LV2), the loading profile indicates that the separation in the LV2 receives positive contributions from Cr, A and Ade and negative contributions from Ala, Gly, choline‐containing compounds, Ac, Val and Lac.

To gain further information, we performed an ANOVA test comparing the deconvoluted areas of 26 metabolites in the three classes of tissue (HC, P and OLP). The metabolites that differ significantly among groups are reported in Table 1: Val, Lac, Ala, Gly, TG, Ser, Glu, glutathione (GSH), Gln and Cho discriminate between HC and P samples and HC and OLP samples. Instead, Oligo (a signal at 5.44 ppm, assigned to 1′‐CH and 1″‐CH of maltose/maltotriose) is only statistically different between HC and OLP samples. No metabolite can discriminate between Ps and OLPs.

**TABLE 1:** Results of the one‐way ANOVA and post hoc test on the deconvoluted areas of the metabolites of the three classes of tissues analysed by HR‐MAS NMR.

	*f*‐value^a^	*p*‐value	−log10(*p*)	FDR^a^	Fisher's LSD^a^	
Val	12.9	0.000058	4.24	0.0015	HC–P	HC–OLP
Lac	7.66	0.0017	2.77	0.020	HC–P	HC–OLP
Ala	7.21	0.0023	2.63	0.020	HC–P	HC–OLP
Gly	6.59	0.0036	2.44	0.022	HC–P	HC–OLP
TG	6.17	0.0050	2.30	0.022	P–HC	OLP–HC
Ser	6.17	0.0050	2.30	0.022	HC–P	HC–OLP
Glu	5.58	0.0077	2.11	0.029	HC–P	HC–OLP
GSH	5.28	0.0097	2.01	0.032	HC–P	HC–OLP
Gln	4.78	0.014	1.84	0.042	HC–P	HC–OLP
Oligo	4.55	0.017	1.76	0.045		HC–OLP
Cho	4.43	0.019	1.72	0.045	HC–P	HC–OLP

Abbreviations: HC, healthy controls; OLP, oral lichen planus; P, perilesional.

^a^ *f*‐value: the ratio between two variables; metabolites with *p* < 0.05 are reported; FDR: false discovery rate accounts for multiple comparisons; Fisher's LSD: Fisher's least significant difference method performs a post‐hoc test in order to identify which classes are different.

**FIGURE 3:**
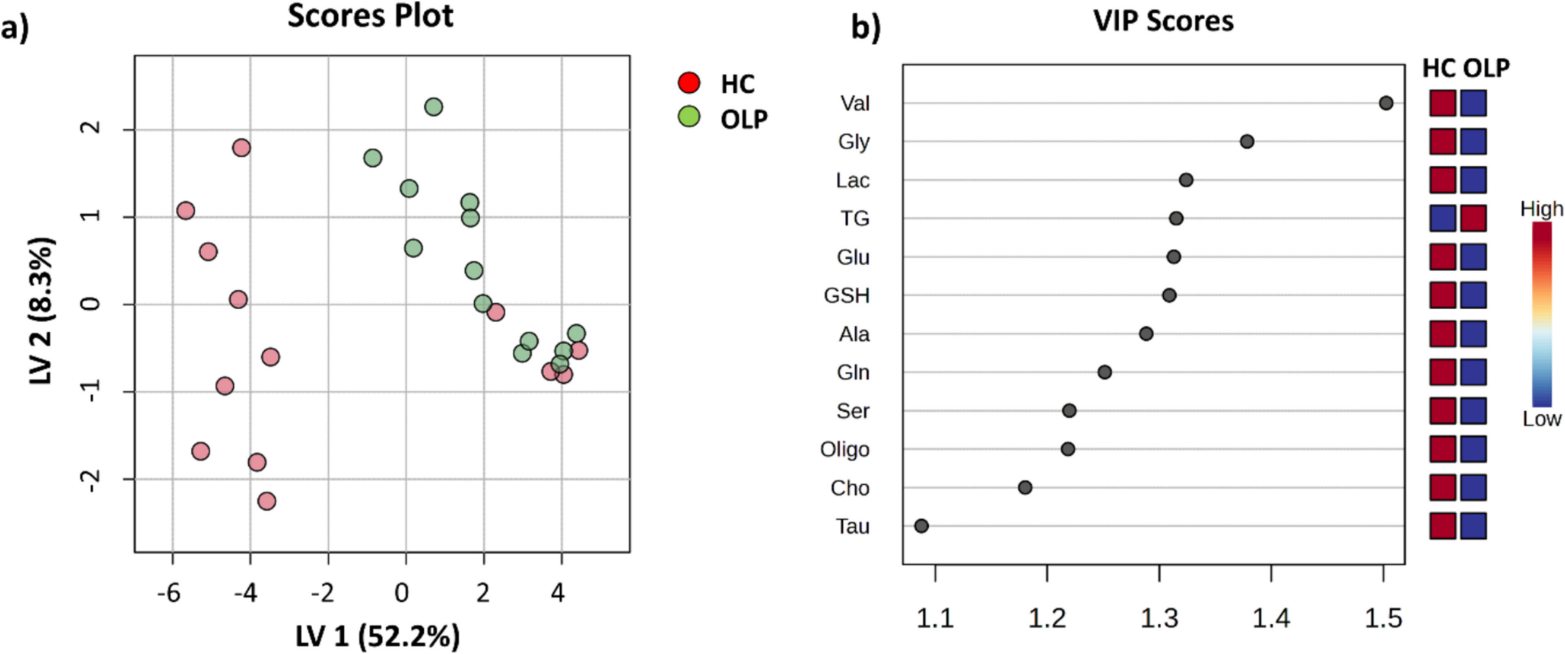
PLS‐DA scores plot (a) and variable importance projection (VIP) scores along LV1 of important tissue metabolites for HC vs. OLP discrimination (b), obtained on deconvoluted ^1^H HR‐MAS CPMG NMR signals of HC (red) and OLP (green) samples. Cross‐validation details are reported in Figure S2.

PLS‐DA analysis was also performed for HCs and OLPs samples Figure 3a. In this case, the separation between the two classes of samples is improved, with respect to that obtained by the spectra analysis; both groups are distinguished, except for four HC samples that display non‐negligible TG signals and that appear together with the OLP samples in the PLS‐DA score plot. Figure 3b highlights a list of relevant metabolites (VIP score > 1) for discriminating HC vs OLP samples. Validation was performed with a five‐fold CV. Details are reported in Figure S2. VIP analysis indicates a higher amount of TG in the OLP samples with respect to HC, whereas the small metabolites Val, Gly, Lac, Glu, GSH, Ala, Gln, Ser, Oligo, Cho and Tau are higher in HC.

### 3.3 ^1^H NMR Analysis of Saliva

We profiled the saliva samples of all study participants (HC and OLP), identifying 68 metabolites, including 27 amino acids and derivatives, 4 simple carbohydrates, 5 purine and pyrimidine‐derived metabolites, 8 compounds from lipid metabolism, 8 organic acids, 2 amines, 11 prokaryotic metabolites and 3 molecules not belonging to any of these groups.

**FIGURE 4:**
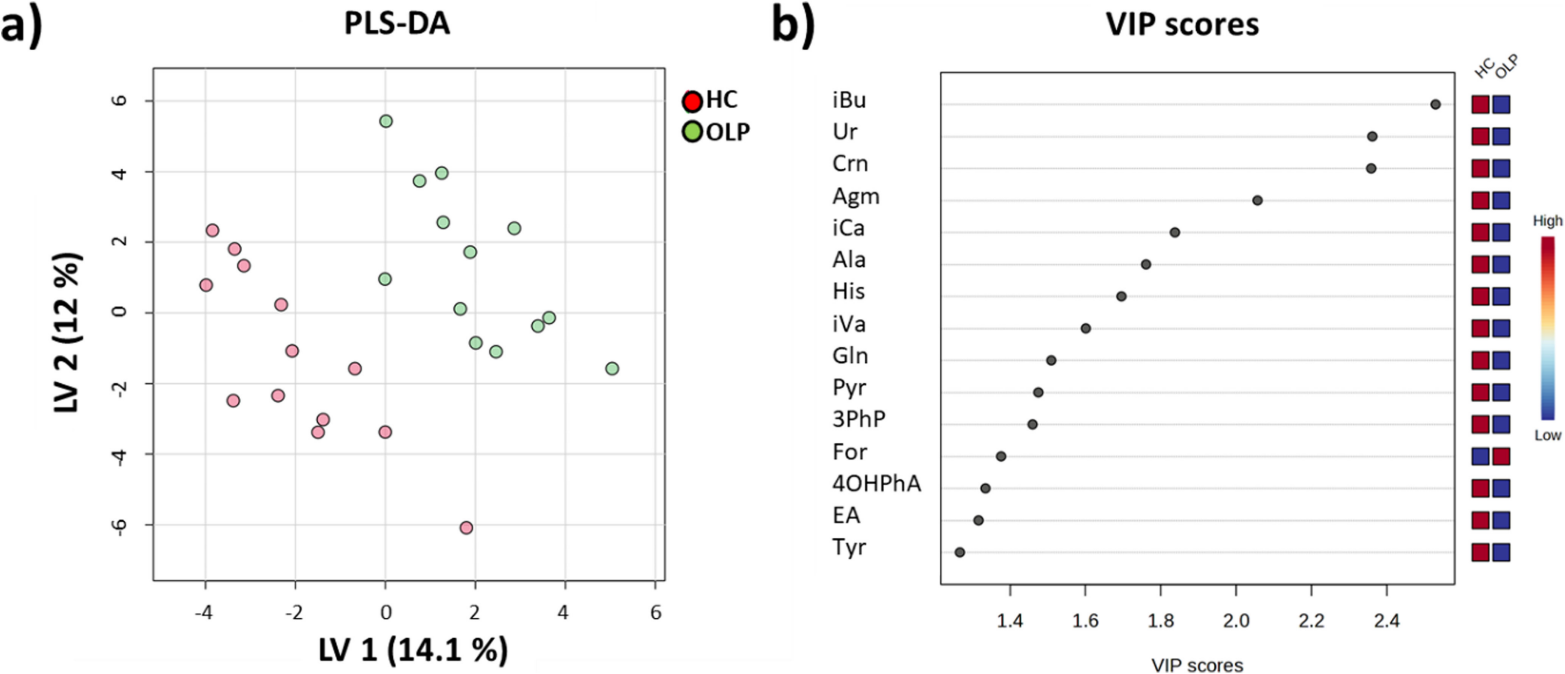
PLS‐DA of salivary metabolic profiles of OLP vs. HC. PLS‐DA 2D score plot (a). PLS‐DA VIP scores (b) depict the 15 most significant metabolites contributing to the OLP and HC separation. The red and blue boxes on the right side of the VIP score plot indicate the relative metabolite abundance in each cluster of the PLS‐DA. 3PhP: 3‐phenylpropionate, 4OHPhA: 4‐hydroxyphenylacetate, Agm: agmatine, Ala: alanine, Crn: creatinine, EA: ethanolamine, For: formate, Gln: glutamine, His: histidine, iBu: isobutyrate, iCa: isocaproate, iVa: isovalerate, Pyr: pyroglutamate, Tyr: tyrosine, Ur: uracil. Cross‐validation details are reported in Figure S3.

PCA based on metabolites' relative concentrations did not show clustering (data not shown). PLS‐DA revealed separate clustering of OLP patients and HCs, with LV1 and LV2 explaining 14.1% and 12% of the total variance between the two groups (Figure 4a). Validation was performed, and details are reported in Figure S3. The compounds driving the separation in the PLS‐DA model are presented in Figure 4b. The most discriminant compounds exhibiting a VIP score > 2 are creatinine (Crn), isobutyrate (iBu), uracil (Ur) and agmatine (Agm).

**FIGURE 5:**
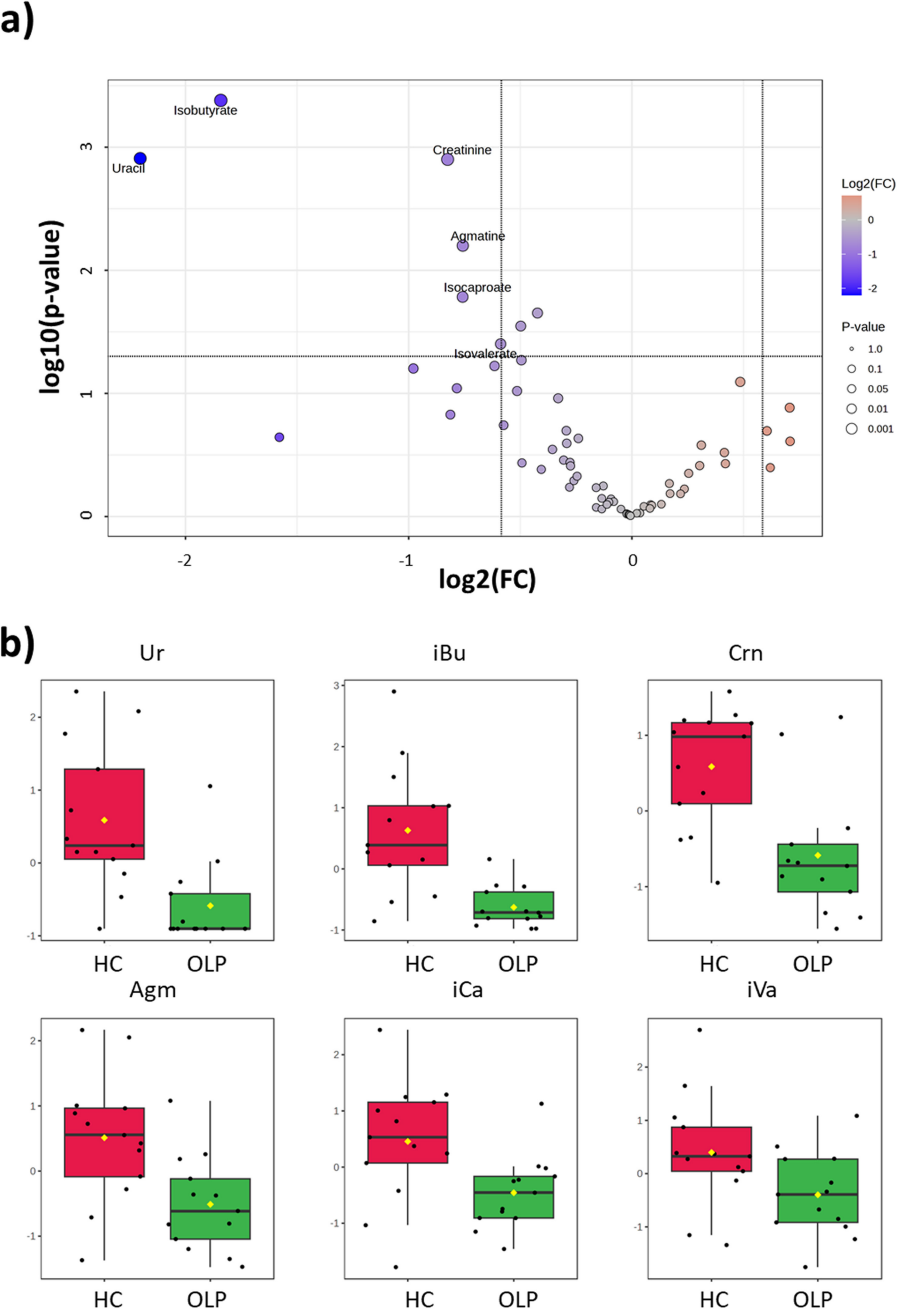
(a) Volcano plot analysis of the differential salivary metabolites of OLP vs. HC. Each point on the volcano plot is based on specific *p* and fold‐change (FC) values. The points that satisfy the condition *p* ≤ 0.05 and |FC| ≥ 1.5 were considered significant and appear in red or blue if the concentration of the corresponding metabolite is higher or lower in OLP than in HC. (b) Box plots of the normalized concentrations of the significant metabolites identified in (a) for both sets of samples; red is for OLP patients, and green is for HC. Agm: agmatine, Crn: creatinine, iBu: isobutyrate, iCa: isocaproate, iVa: isovalerate, Ur: uracil.

The univariate volcano plot analysis further validated the statistically significant compounds. By setting the fold change threshold at 1.5 and a *p*‐value ≤ 0.05 (Figure 5a), Ur, iBu, Crn, Agm, isocaproate (iCa) and isovalerate (iVa) appear more concentrated in HCs. The box plots in Figure 5b show the distribution of the normalized concentrations of the six most significant metabolites responsible for discriminating between HC and OLP samples.

## 4 Discussion

Uncovering the precise pathogenesis of OLP and defining reliable strategies for monitoring treatment efficacy and potentially malignant changes remain among the major challenges of oral medicine. Despite more than 50 years of active research, this disease still represents a dilemma mainly with regard to onset, progression and prognosis. To the best of our knowledge, a thorough metabolomic evaluation through ^1^H NMR spectroscopy combining oral tissues and saliva from patients affected by OLP has never been attempted before.

Even though the limitations of the present study (e.g., small number of cases and selection of patients with reticular OLP pattern) may allow us to draw only provisional hypotheses, the findings reported here could be of paramount importance, particularly if further studies include cases of erosive disease as well as dysplastic lesions. Our cohort was deliberately restricted to bilateral reticular lesions to reduce biological variability; however, this choice limits generalizability to other clinical variants. Erosive and ulcerative OLP—typically associated with a greater inflammatory burden—may show distinct metabolic signatures. Future cross‐subtype analyses are required to define the span of these biomarkers. On another level, the extremely complex interaction between healthy mucosa, areas of inflammatory diseases and microbial species could be somewhat enlightened by the identification and correlations of changes in metabolite concentration in oral fluids and tissues.

Statistical analysis of salivary metabolite concentrations revealed significant changes in some metabolites of bacterial origin, such as iBu, iCa, iVa and Agm, suggesting that OLP lesions are possibly associated with an imbalance in microbiota composition. This finding has clinical implications, as microbiome‐targeted therapies could represent promising adjunctive treatments. Improved oral hygiene practices and periodic periodontal assessments might further support clinical management by reducing microbial‐induced inflammation. Ur, together with iBu, iVa and iCa, is found only in whole saliva [27] and reflects the metabolic interaction between the salivary glands' secretions, gingival crevicular fluid, suspended bacteria, desquamated cells and food debris. Agm, a derivative of arginine, can be produced by plaque bacteria that express the enzyme arginine decarboxylase (ADC) [28]. Instead, blood‐derived molecules, such as Crn, can also enter the saliva via the highly vascularized salivary glands and may be associated with systemic diseases that require special attention from health professionals [27, 29]. Regular tracking of these metabolic alterations could enable clinicians to identify periods of exacerbation and remission, potentially allowing timely interventions to mitigate clinical outcomes such as gingival damage as well as severe mucosal erosion.

In ^1^H HR‐MAS NMR analysis of tissues, TG were identified as the metabolites that discriminate both P and OLP, with higher TG levels from healthy tissue. In addition, other metabolites such as Glu, Ser, Tau, Lac, GSH, Cho, Val, Ala and Gly were also significant, with reduced concentrations in OLP and P tissues compared with healthy tissue. Most of the cited metabolites are amino acids that are important units of energy sources for basic metabolic pathways in human beings. The higher levels of TG in OLP patients than in healthy subjects may be related to increased inflammation and immune dysfunction. It is known that OLP pathogenesis depends on alterations of immune function and inflammation [1, 30]. The high amount of TG may inhibit the production of inflammatory cytokines, such as TNF‐α, and, in turn, influence its downstream activities, including the inhibition of NF‐κB [31]. NF‐κB plays a key role in cellular inflammation and immune response [32]. The dysregulation of NF‐κB may cause autoimmune diseases, chronic inflammation and even cancer [33, 34]. Studies have shown that TNF‐α is overexpressed in OLP patients, and the metabolic alterations of TG and omega‐3 may be related to the elevated expression of TNF‐α and activation of the NF‐κB signalling pathway. These findings highlight the potential for adjunctive immunomodulatory therapies beyond conventional topical corticosteroids, specifically targeting lipid metabolism or inflammatory pathways.

The tissue content of Glu and Gln is decreased in the OLP group. Glu is thought to be an important regulator of oxidative stress [35]. Oxidative stress associated with inflammation exacerbates the onset and progression of OLP [36, 37]. Accordingly, typical Glu metabolism may induce oxidative stress, leading to OLP. Previous studies suggest that most patients with OLP are affected by emotional stress, such as insomnia and anxiety and that mental factors may promote the onset of regulatory genes involved in the biosynthetic pathways and regulatory mechanisms of metabolites in oxidative stress. The recognized role of glutamate dehydrogenase 1 (GLUD1) is a consequence of the efficient transfer by transaminases of the α‐amino group of several amino acids to 2‐oxoglutarate, forming Glu [38]. Therefore, the decrease in GLUD1 level may be one of the direct reasons for the decrease in Glu content in OLP tissue. The mRNA level of glutamine‐fructose‐6‐phosphate transaminase 2 (GFPT2) is upregulated in many cancers, such as glioblastoma, lung adenocarcinoma and breast cancer [39, 40]. Xin and coworkers report that the level of GFPT2 is upregulated in patients with OLP, which may be the regulatory mechanism associated with the malignant transformation of OLP [41, 42, 43]. We found a decrease in Tau in this study, although only at a *p*‐level of 0.070. GSH plays a role in oxidative stress by regulating the redox state and immune response [44], and Tau is also closely associated with the development of the immune system [45]. It has been suggested that OLP patients might have immune system dysfunction since a significantly reduced level of Tau has been observed in them. This could be a potential etiological factor of OLP.

**FIGURE 6:**
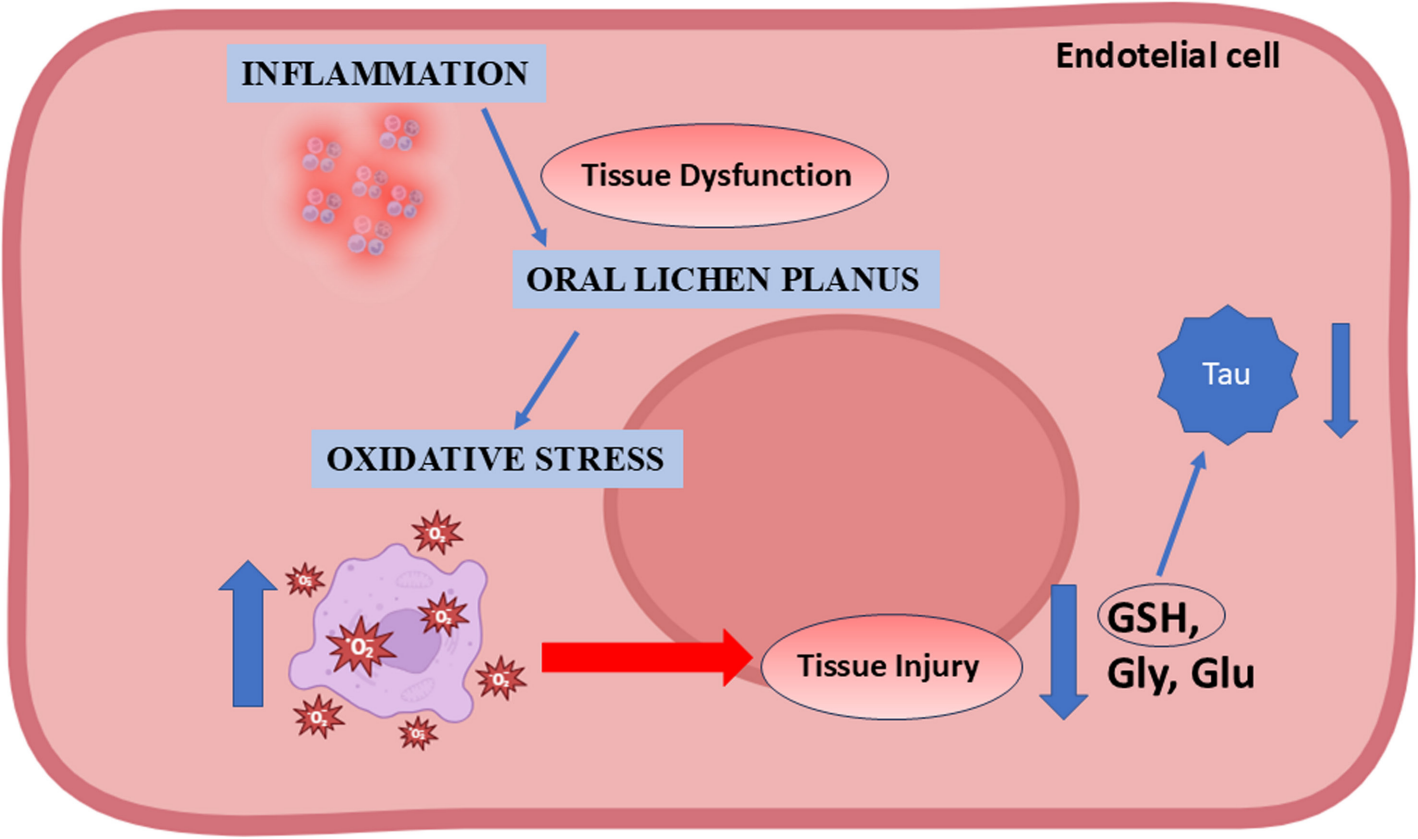
Hypothesis of a ROS‐mediated mechanism in OLP disease.

The reduction of the highlighted metabolites and the increase of TG are associated with the increase of ROS and oxidative damage. We can hypothesize that they are closely related to the occurrence and development of OLP [46], together with increased lipid peroxidation and an imbalance of the antioxidant defence system (Figure 6). Such insights advocate for antioxidant‐based therapeutic interventions aimed at attenuating oxidative damage and possibly slowing OLP progression.

In conclusion, although the composition of salivary metabolites has suggested an alteration of the oral microbiota, metabolites peculiar to OLP lesions supported the hypothesis based on increased lipid peroxidation and an imbalance in the antioxidant defence to explain the occurrence and development of OLP. Indeed, based on NMR spectroscopy analysis, several metabolites revealed their potential to differentiate OLP from HC, which may have important clinical implications. Hence, metabolomic profiles could potentially be used to diagnose or monitor OLP progression and response to therapy, helping to unify decision‐making across different specialties. Combined with clinical parameters such as lesion morphology and symptom severity, metabolomics could facilitate a personalized approach to OLP care. Although the risk of malignant transformation in OLP is relatively low, identifying patients most at risk remains a clinical challenge. Metabolomic markers that shift significantly in more aggressive or persistent lesions might serve as ‘red flags’. For instance, a persistent increase in TG or further depletion of key antioxidants (e.g., GSH) could signal a higher risk profile. Changes in salivary or tissue metabolite profiles over time could prompt earlier biopsy or closer follow‐up, allowing for early intervention.

To our knowledge, this is the first HR‐MAS NMR study of OLP tissue combined with salivary metabolomics. It reveals elevated levels of tissue TG associated with oxidative stress, and increased concentrations of specific bacterial‐derived organic acids in saliva that discriminate OLP from controls. These findings extend prior saliva‐only studies by pinpointing metabolic pathways at the lesion site and suggesting minimally invasive biomarkers. The interpretation of these findings in the context of clinical variability and disease progression remains challenging. Further research must address these biochemical–clinical relationships more explicitly, potentially leveraging longitudinal designs and comprehensive clinical scoring systems. The identification of novel biomarkers and biochemical pathways underscores exciting possibilities for enhancing diagnostic accuracy and guiding individualized treatments. As metabolomics techniques become more accessible, they stand to play a pivotal role in shaping future OLP patient management strategies. Finally, if these findings are validated in a large‐scale study with larger cohorts of volunteers enabling more robust statistical analyses, the integration of histopathological assessment with NMR of tissues, at the diagnostic stage (or when a second biopsy is already clinically indicated), and non‐invasive saliva NMR screening for routine surveillance could be considered for clinical applications.

## Conflicts of Interest

The authors declare no conflicts of interest.

## 5 Supporting Information

The following Supporting Information is available: Table S1. ^1^H and ^13^C NMR Chemical Shifts of the identified metabolites. Figure S1. PLS‐DA scores plot and LV1 and LV2 loadings obtained from ^1^H HR‐MAS NMR CPMG spectra of tissue samples. Table S2. PLS‐DA cross‐validation details from deconvoluted HR‐MAS NMR data. Table S3. PLS‐DA cross‐validation details from saliva NMR data

## Supporting information


**Table S1:** ^1^H and ^13^C NMR chemical shifts of the metabolites identified in tissue.
**Figure S1:** PLS‐DA scores plot and LV1 and LV2 loadings obtained from ^1^H HR‐MAS NMR CPMG spectra.
**Figure S2:** PLS‐DA cross‐validation details from deconvoluted HR‐MAS NMR data obtained from tissues.
**Figure S3:** PLS‐DA cross‐validation details from saliva NMR data.

## Data Availability

The deconvoluted signals matrixes from saliva and oral mucosa biopsies have been deposited in the IRIS repository (https://iris.unimore.it/), with ID: d2a5a37e‐17be‐49c2‐a957‐2986bbe30e84; they can be requested to the authors via the link: https://hdl.handle.net/11380/1377108.

## References

[1] C. Scully and M. Carrozzo , “Oral Mucosal Disease: Lichen Planus,” British Journal of Oral & Maxillofacial Surgery 46 (2008): 15–21, 10.1016/j.bjoms.2007.07.199. 17822813

[2] M. Carrozzo , “Understanding the Pathobiology of Oral Lichen Planus,” Current Oral Health Reports 1, no. 3 (2014): 173–179, 10.1007/s40496-014-0022-y.

[3] M. Carrozzo , “A Personal Journey Through Oral Medicine: The Tale of Hepatitis C Virus and Oral Lichen Planus,” Journal of Oral Pathology & Medicine 52 (2023): 335–338, 10.1111/jop.13400. 36597838

[4] S. Warnakulasuriya , “Clinical Features and Presentation of Oral Potentially Malignant Disorders,” Oral Surgery, Oral Medicine, Oral Pathology and Oral Radiology 125 (2018): 582–590, 10.1016/j.oooo.2018.03.011. 29673799

[5] R. M. López‐Pintor , M. Diniz‐Freitas , S. S. K. Ramesh , et al., “World Workshop on Oral Medicine VIII: Development of a Core Outcome Set for Oral Lichen Planus: A Systematic Review of Outcome Domains,” Oral Surgery, Oral Medicine, Oral Pathology and Oral Radiology 135 (2023): 772–780, 10.1016/j.oooo.2023.01.014. 37061409

[6] C. Li , X. Tang , X. Zheng , et al., “Global Prevalence and Incidence Estimates of Oral Lichen Planus: A Systematic Review and Meta‐Analysis,” JAMA Dermatology 156 (2020): 172–181, 10.1001/jamadermatol.2019.3797. 31895418 PMC6990670

[7] M. Á. González‐Moles , I. Ruiz‐Ávila , L. González‐Ruiz , Á. Ayén , J. A. Gil‐Montoya , and P. Ramos‐García , “Malignant Transformation Risk of Oral Lichen Planus: A Systematic Review and Comprehensive Meta‐Analysis,” Oral Oncology 96 (2019): 121–130, 10.1016/j.oraloncology.2019.07.012. 31422203

[8] X. Cai , J. Zhang , H. Zhang , and T. Li , “Overestimated Risk of Transformation in Oral Lichen Planus,” Oral Oncology 133 (2022): 106025, 10.1016/j.oraloncology.2022.106025. 35858493

[9] A. El‐Howati , M. H. Thornhill , H. E. Colley , and C. Murdoch , “Immune Mechanisms in Oral Lichen Planus,” Oral Diseases 29 (2023): 1400–1415, 10.1111/odi.14142. 35092132

[10] N. Gururaj , P. Hasinidevi , V. Janani , and T. Divynadaniel , “Diagnosis and Management of Oral Lichen Planus—Review,” Journal of Oral and Maxillofacial Pathology 25 (2021): 383–393, 10.4103/jomfp.jomfp_386_21. 35281147 PMC8859620

[11] S. Gupta , S. Ghosh , and S. Gupta , “Interventions for the Management of Oral Lichen Planus: A Review of the Conventional and Novel Therapies,” Oral Diseases 23 (2017): 1029–1042, 10.1111/odi.12634. 28055124

[12] X. Wang , L. Liu , Q. Du , et al., “Human Saliva Metabolome for Oral Lichen Planus Biomarker Identification,” Recent Patents on Anti‐Cancer Drug Discovery 16 (2021): 417–425, 10.2174/1574892816666210224160120. 33655848

[13] G. Setti , V. Righi , A. Mucci , et al., “Metabolic Profile of Whole Unstimulated Saliva in Patients With Sjogren's Syndrome,” Metabolites 13 (2023): 348, 10.3390/metabo13030348. 36984788 PMC10054882

[14] M. Meleti , E. Quartieri , R. Antonelli , et al., “Metabolic Profiles of Whole, Parotid and Submandibular/Sublingual Saliva,” Metabolites 10 (2020): 318, 10.3390/metabo10080318. 32781584 PMC7466076

[15] X. Y. Yang , X. Z. Li , and S. N. Zhang , “Metabolomics Analysis of Oral Mucosa Reveals Profile Perturbation in Reticular Oral Lichen Planus,” Clinica Chimica Acta 487 (2018): 28–32, 10.1016/j.cca.2018.09.021. 30218656

[16] B. Kashyap , E. Hyvärinen , I. Laitinen , and A. M. Kullaa , “Salivary Metabolomics in Patients With Oral Lichen Planus: A Preliminary Study Based on NMR Spectroscopy,” Clinical Oral Investigations 28 (2024): 103, 10.1007/s00784-023-05389-1. 38236502 PMC10796579

[17] J. Figueira , S. Gouveia‐Figueira , C. Öhman , P. L. Holgerson , M. L. Nording , and A. Öhman , “Metabolite Quantification by NMR and LC‐MS/MS Reveals Differences Between Unstimulated, Stimulated, and Pure Parotid Saliva,” Journal of Pharmaceutical and Biomedical Analysis 140 (2017): 295–300, 10.1016/j.jpba.2017.03.037. 28380387

[18] A. Gardner , H. G. Parkes , G. H. Carpenter , and P. W. So , “Developing and Standardizing a Protocol for Quantitative Proton Nuclear Magnetic Resonance (1H NMR) Spectroscopy of Saliva,” Journal of Proteome Research 17 (2018): 1521–1531, 10.1021/acs.jproteome.7b00847. 29498859 PMC6558279

[19] S. Kumari , V. Goyal , S. S. Kumaran , S. N. Dwivedi , A. Srivastava , and N. R. Jagannathan , “Quantitative Metabolomics of Saliva Using Proton NMR Spectroscopy in Patients With Parkinson's Disease and Healthy Controls,” Neurological Sciences 41 (2020): 1201–1210, 10.1007/s10072-019-04143-4. 31897951

[20] J. L. Pereira , D. Duarte , T. J. Carneiro , et al., “Saliva NMR Metabolomics: Analytical Issues in Pediatric Oral Health Research,” Oral Diseases 25 (2019): 1545–1554, 10.1111/odi.13117. 31077633

[21] S. Wallner‐Liebmann , L. Tenori , A. Mazzoleni , et al., “Individual Human Metabolic Phenotype Analyzed by ^1^H NMR of Saliva Samples,” Journal of Proteome Research 15 (2016): 1787–1793, 10.1021/acs.jproteome.5b01060. 27087681

[22] L. Schenetti , A. Mucci , F. Parenti , et al., “HR‐MAS NMR Spectroscopy in the Characterization of Human Tissues: Application to Healthy Gastric Mucosa,” Concepts in Magnetic Resonance Part A 28A (2006): 430–443, 10.1002/cmr.a.20068.

[23] E. Quartieri , E. Casali , E. Ferrari , et al., “Sample Optimization for Saliva ^1^H‐NMR Metabolic Profiling,” Analytical Biochemistry 640 (2022): 114412, 10.1016/j.ab.2021.114412. 34656613

[24] M. Gallo , S. Matteucci , N. Alaimo , et al., “A Novel Method Using Nuclear Magnetic Resonance for Plasma Protein Binding Assessment in Drug Discovery Programs,” Journal of Pharmaceutical and Biomedical Analysis 167 (2019): 21–29, 10.1016/j.jpba.2019.01.049. 30738240

[25] M. Gallo , L. Giovati , W. Magliani , et al., “Metabolic Plasticity of *Candida albicans* in Response to Different Environmental Conditions,” Journal of Fungi 8 (2022): 723, 10.3390/jof8070723. 35887478 PMC9322845

[26] Z. Pang , J. Chong , G. Zhou , et al., “MetaboAnalyst 5.0: Narrowing the Gap Between Raw Spectra and Functional Insights,” Nucleic Acids Research 49 (2021): W388–W396, 10.1093/nar/gkab382. 34019663 PMC8265181

[27] E. Ferrari , M. Gallo , A. Spisni , R. Antonelli , M. Meleti , and T. A. Pertinhez , “Human Serum and Salivary Metabolomes: Diversity and Closeness,” International Journal of Molecular Sciences 24 (2023): 16603, 10.3390/ijms242316603. 38068926 PMC10706786

[28] M. M. Nascimento and R. A. Burne , “Caries Prevention by Arginine Metabolism in Oral Biofilms: Translating Science Into Clinical Success,” Current Oral Health Reports 1 (2014): 79–85, 10.1007/s40496-013-0007-2.

[29] J. Cassol‐Spanemberg , M. E. Rodríguez‐de Rivera‐Campillo , E. M. Otero‐Rey , A. Estrugo‐Devesa , E. Jané‐Salas , and J. López‐López , “Oral Lichen Planus and Its Relationship With Systemic Diseases. A Review of Evidence,” Journal of Clinical and Experimental Dentistry 10 (2018): e938–e944, 10.4317/jced.55145. 30386529 PMC6203921

[30] M. D. Mignogna , S. Fedele , L. Lo Russo , L. Lo Muzio , and E. Bucci , “Immune Activation and Chronic Inflammation as the Cause of Malignancy in Oral Lichen Planus: Is There Any Evidence?,” Oral Oncology 40 (2004): 120–130, 10.1016/j.oraloncology.2003.08.001. 14693234

[31] Y. Zhao , S. Joshi‐Barve , S. Barve , and L. H. Chen , “Eicosapentaenoic Acid Prevents LPS‐Induced TNF‐Alpha Expression by Preventing NF‐KappaB Activation,” Journal of the American College of Nutrition 23 (2004): 71–78, 10.1080/07315724.2004.10719345. 14963056

[32] K. M. Pflug and R. Sitcheran , “Targeting NF‐kappaB‐Inducing Kinase (NIK) in Immunity, Inflammation, and Cancer,” International Journal of Molecular Sciences 21 (2020): 8470, 10.3390/ijms21228470. 33187137 PMC7696043

[33] J. Wang , X. Zhai , J. Guo , et al., “Long Non‐Coding RNA DQ786243 Modulates the Induction and Function of CD4+ Treg Cells Through Foxp3‐miR‐146a‐NF‐κB Axis: Implications for Alleviating Oral Lichen Planus,” International Immunopharmacology 75 (2019): 105761, 10.1016/j.intimp.2019.105761. 31325726

[34] A. Soleimani , F. Rahmani , G. A. Ferns , M. Ryzhikov , A. Avan , and S. M. Hassanian , “Role of the NF‐KappaB Signaling Pathway in the Pathogenesis of Colorectal Cancer,” Gene 726 (2020): 144132, 10.1016/j.gene.2019.144132. 31669643

[35] W. Tang , J. Wu , S. Jin , et al., “Glutamate and Aspartate Alleviate Testicular/Epididymal Oxidative Stress by Supporting Antioxidant Enzymes and Immune Defense Systems in Boars,” Science China. Life Sciences 63 (2020): 116–124, 10.1007/s11427-018-9492-8. 31102177

[36] S. Banerjee , S. Mukherjee , S. Mitra , and P. Singhal , “Comparative Evaluation of Mitochondrial Antioxidants in Oral Potentially Malignant Disorders,” Kurume Medical Journal 66 (2019): 15–27, 10.2739/kurumemedj.MS661009. 32378537

[37] M. Wu , H. Xiao , W. Ren , et al., “Therapeutic Effects of Glutamic Acid in Piglets Challenged With Deoxynivalenol,” PLoS ONE 9 (2014): e100591, 10.1371/journal.pone.0100591. 24984001 PMC4077692

[38] R. Moreno‐Sánchez , Á. Marín‐Hernández , J. C. Gallardo‐Pérez , et al., “Physiological Role of Glutamate Dehydrogenase in Cancer Cells,” Frontiers in Oncology 10 (2020): 429, 10.3389/fonc.2020.00429. 32328457 PMC7160333

[39] U. Verbovšek , H. Motaln , A. Rotter , et al., “Expression Analysis of All Protease Genes Reveals Cathepsin K to be Overexpressed in Glioblastoma,” PLoS ONE 9 (2014): e111819, 10.1371/journal.pone.0111819. 25356585 PMC4214761

[40] W. Zhang , G. Bouchard , A. Yu , et al., “GFPT2‐Expressing Cancer‐Associated Fibroblasts Mediate Metabolic Reprogramming in Human Lung Adenocarcinoma,” Cancer Research 78 (2018): 3445–3457, 10.1158/0008-5472.CAN-17-2928. 29760045 PMC6030462

[41] M. Z. Xin , Y. Y. Shi , C. S. Li , et al., “Metabolomics and Transcriptomics Analysis on Metabolic Characteristics of Oral Lichen Planus,” Frontiers in Oncology 11 (2021): 769163, 10.3389/fonc.2021.769163. 34737967 PMC8560742

[42] X. S. Wang , Z. Sun , L. W. Liu , et al., “Potential Metabolic Biomarkers for Early Detection of Oral Lichen Planus, a Precancerous Lesion,” Frontiers in Pharmacology 11 (2020): 603899, 10.3389/fphar.2020.603899. 33240093 PMC7677577

[43] X. Li , L. Liu , N. Li , et al., “Metabolomics Based Plasma Biomarkers for Diagnosis of Oral Squamous Cell Carcinoma and Oral Erosive Lichen Planus,” Journal of Cancer 13 (2022): 76–87, 10.7150/jca.59777. 34976172 PMC8692701

[44] J. Piao , F. Meng , H. Fang , et al., “Effect of Taurine on Thymus Differentiation of Dex‐Induced Immunosuppressive Mice,” Advances in Experimental Medicine and Biology 1155 (2019): 381–390, 10.1007/978-981-13-8023-5_36. 31468416

[45] Y. Mu , Y. Zhou , Y. Wang , et al., “Serum Metabolomics Study of Nonsmoking Female Patients With Non‐Small Cell Lung Cancer Using Gas Chromatography‐Mass Spectrometry,” Journal of Proteome Research 18 (2019): 2175–2184, 10.1021/acs.jproteome.9b00069. 30892048

[46] F. H. Panchal , S. Ray , R. P. Munshi , S. S. Bhalerao , and C. S. Nayak , “Alterations in Lipid Metabolism and Antioxidant Status in Lichen Planus,” Indian Journal of Dermatology 60 (2015): 439–444, 10.4103/0019-5154.159624. 26538688 PMC4601408

