# Supplementary material for: Tissue and Salivary NMR Metabolomics in Reticular‐Type Oral Lichen Planus
Source: NMR Biomed. 2025 Sep 3;38(10):e70137. doi: 10.1002/nbm.70137 (PMC12409093; doi:10.1002/nbm.70137)
Supplement: Supplementary file 1 — Table S1: 1H and 13C NMR chemical shifts of the metabolites identified in tissue. Figure S1: PLS‐DA scores plot and LV1 and LV2 loadings obtained from 1H HR‐MAS NMR CPMG spectra. Figure S2: PLS‐DA cross‐validation details from deconvoluted HR‐MAS NMR data obtained from tissues. Figure S3: PLS‐DA cross‐validation details from saliva NMR data. [file NBM-38-e70137-s001.docx]

**SUPPLEMENTARY MATERIAL**

**Tissue and Salivary NMR Metabolomics in Reticular-Type Oral Lichen Planus**

Giacomo Setti†, Anna Gambini†, Valeria Righi*, Adele Mucci*, Thelma A. Pertinhez, Elena Ferrari, Mariana Gallo, Brunella Biscussi, Rita Antonelli, Marco Meleti, Cristina Magnoni

**Summary**

**Table S1**. ^1^H and ^13^C NMR Chemical Shifts of the metabolites identified in tissue p 2-5

**Figure S1**. PLS-DA scores plot and LV1 and LV2 loadings obtained from ^1^H HR-MAS NMR CPMG spectra p 6

**Figure S2**. PLS-DA cross-validation details from deconvoluted HR-MAS NMR data obtained from tissues p 7

**Figure S3**. PLS-DA cross-validation details from saliva NMR data p 7

**Table S1**. ^1^H and ^13^C NMR Chemical Shift of the metabolites^a^ identified in oral mucosa biopsies

|  | **Metabolites** | **δ^1^H**^a^ | **δ^13^C^b^** | **Assignment** |
| --- | --- | --- | --- | --- |
| **1** | FA | 0.91 | 16.9 | CH_3_ |
|  |  | 0.98 | 16.9 | CH_3_ (ω-3) |
|  |  | 1.37–1.29 | 32.4 | (CH_2_)_n_ |
|  |  | 1.60 | 27.7 | CH_2_-CH_2_-C=O |
|  |  | 2.08 | 22.6 | CH_3_CH_2_CH=CH((ω-3) |
|  |  | 2.07–2.04 | 29.8 | CH_2_CH=CH |
|  |  | 2.27 | 36.5 | CH_2_-C=O |
|  |  | 2.81-2.78 | 28.4 | =CH-CH_2_-CH= |
|  |  | 5.30-5.33 | 130.7,132.5 | -CH=CH- |
| **2** | Leu | 0.96 (d) | 23.6 | δ-CH_3_ |
|  |  | 0.97 (d) | 24.8 | δ-CH_3_ |
|  |  | 1.71 |  | β-CH_2_ |
|  |  | 1.72 |  | γ-CH |
|  |  |  |  | α-CH |
| 3 | Ile | 0.93 (t) |  | δ-CH_3_ |
|  |  | 1.01 (d) |  | γ-CH_3_ |
|  |  | 1.25 |  | γ'-CH_3_ |
|  |  | 1.45 |  | γ'-CH_3_ |
|  |  | 1.96 |  | β-CH |
|  |  | 3.65 |  | α-CH |
| **4** | Val | 0.99 (d) | 19.4 | γ-CH_3_ |
|  |  | 1.04 (d) | 20.6 | γ-CH_3_ |
|  |  | 2.26 |  | β-CH |
|  |  | 3.60 |  | α-CH |
| **5** | Lac | 1.33 (d) | 23.0 | CH_3_ |
|  |  | 4.11 (q) | 71.3 | CH |
| **6** | Ala | 1.48 | 19.0 | CH_3_ |
|  |  | 3.78 |  | α-CH |
| **7** | Ac | 1.92 (s) |  | CH_3_ |
| **8** | Glu | 2.13, 2.07 |  | β-CH_2_ |
|  |  | 2.35 | 36.3 | γ-CH_2_ |
|  |  | ^c^ |  | α-CH |
| **9** | Gln | 2.14 |  | β-CH_2_ |
|  |  | 2.44 | 33.8 | γ-CH_2_ |
|  |  | ^c^ |  | α-CH |
| 10 | GSH | 2.15 |  | β-CH_2glu_ |
|  |  | 2.56 |  | γ-CH_2glu_ |
|  |  | ^c^ |  | α-CH |
|  |  | 2.95 |  | β-CH_2cys_ |
|  |  | 4.58 |  | α-CH_cys_ |
|  |  | nd |  | CH_2gly_ |
| **11** | Asp | 2.82, 2.68 |  | β-CH_2_ |
|  |  | 3.89 |  | α-CH |
| **12** | Lys | nd |  | γ-CH_2_ |
|  |  | 1.72 | 29.4 | δ-CH_2_ |
|  |  | 1.91 | 32.8 | β-CH_2_ |
|  |  | 3.02 | 41.9 | ε-CH_2_ |
|  |  | 3.76 | ^c^ | α-CH |
|  |  |  |  |  |
| **13** | Cr | 3.03 (s) | 39.8 | CH_3_ |
|  |  | 3.94 (s) | 56.5 | CH_2_ |
| **14** | Pro | 2.01 |  | γ-CH_2_ |
|  |  | 2.34,2.05 |  | β-CH_2_ |
|  |  | 3.40,3.31 |  | δ-CH_2_ |
|  |  | 4.13 |  | α-CH |
| **15** | EtOH | 1.18 (t) |  |  |
|  |  | 3.65 |  |  |
| **16** | Cho | 3.20 (s) | 56.7 | N(CH_3_)_3_ |
|  |  | 3.50 |  | NCH_2_ |
|  |  | 4.06 |  | OCH_2_ |
| **17** | PC | 3.23 (s) | 56.7 | N(CH_3_)_3_ |
|  |  | 3.61 |  | NCH_2_ |
|  |  | 4.17 |  | OCH_2_ |
| **18** | GPC | 3.24 (s) | 56.8 | N(CH_3_)_3_ |
|  |  | 3.69 |  |  |
|  |  | 4.33 |  |  |
| **19** | Tau | 3.26 (t) | 50.4 | S-CH_2_ |
|  |  | 3.42 (t) | 38.3 | N-CH_2_ |
| **20** | Gly | 3.56 (s) | 44.4 | CH_2_ |
| **21** | β-Glc | 3.25 |  | 2-CH |
|  |  | 3.40 |  | 4-CH |
|  |  | 3.47 |  | 5-CH |
|  |  | 3.50 |  | 3-CH |
|  |  | 3.90, 3.73 |  | 6-CH_2_ |
|  |  | 4.65 (d) |  | 1-CH |
| **22** | α-Glc | 3.42 |  | 4-CH |
|  |  | 3.53 |  | 2-CH |
|  |  | 3.71 |  | 3-CH |
|  |  | 5.24 (d) |  | 1-CH |
| **23** | Oligo | 5.44 |  | 1'-CH and 1''-CH |
| **24** | U | 7.90 |  | 6-CH |
|  |  | 5.90 |  | 5-CH |
|  |  | 5.92 |  | 1'-CH |
|  |  | 4.33 |  | 2'-CH |
| **25** | Ur | 7.54 |  | 6-CH |
|  |  | 5.80 |  | 5-CH |
| **26** | Asc | 4.52 |  | 4-CH |
|  |  | 4.02 |  | 5-CH |
| **27** | Suc | 2.41 |  | CH_2_ |
| **28** | Ser | 3.85 |  | α-CH |
|  |  | 3.95, 3.98 |  | β-CH_2_ |
| **29** | Ade | 8.19 |  | 8-CH |
| **30** | A | 8.36 |  | 8-CH |
|  |  | 6.09 |  | 1'-CH ribose |
| **31** | glycerol bound in TG | 4.31, 4.10 | 64.6 | CH_2_ |
|  |  | 5.24 | 71.7 | CH |
| **32** | F | 8.45 |  | HCOOH |
| **33** | Tyr | 6.89 |  | 3,5-CH |
|  |  | 7.18 |  | 2,6-CH |
| **34** | Phe | 7.32 |  | H-*o* |
|  |  | 7.37 |  | H-*p* |
|  |  | 7.42 |  | H-*m* |
| **35** | phosphoethanolamine | 3.22 | 43.6 | CH_2_N |
|  |  | 3.98 | 63.0 | CH_2_O |
| **36** | Scy | 3.35 |  | CH |
| **37** | MeOH | 3.36 | 51.6 | CH_3_ |
| **38** | articaine | 7.50 |  | CH (thiophene) |
|  |  | 4.25 |  | NCH |
|  |  | 3.85 |  | OCH_3_ |
|  |  | 3.10-3.01 |  | NCH_2_ |
|  |  | 2.10 |  | CH_3_ (thiophene) |
|  |  | 1.74 |  | -CH_2_CH_3_ |
|  |  | 1.71 |  | -CHCH_3_ |
|  |  | 1.00 |  | -CH_2_CH_3_ |

^a^ ^1^H chemical shifts refer to Ala doublet at 1.48 ppm;

^b^ ^13^C chemical shifts refer to Ala at 19.0 ppm.

^c^ contributes to the 3.77, 57.2 ppm cross-peak in HSQC spectra.


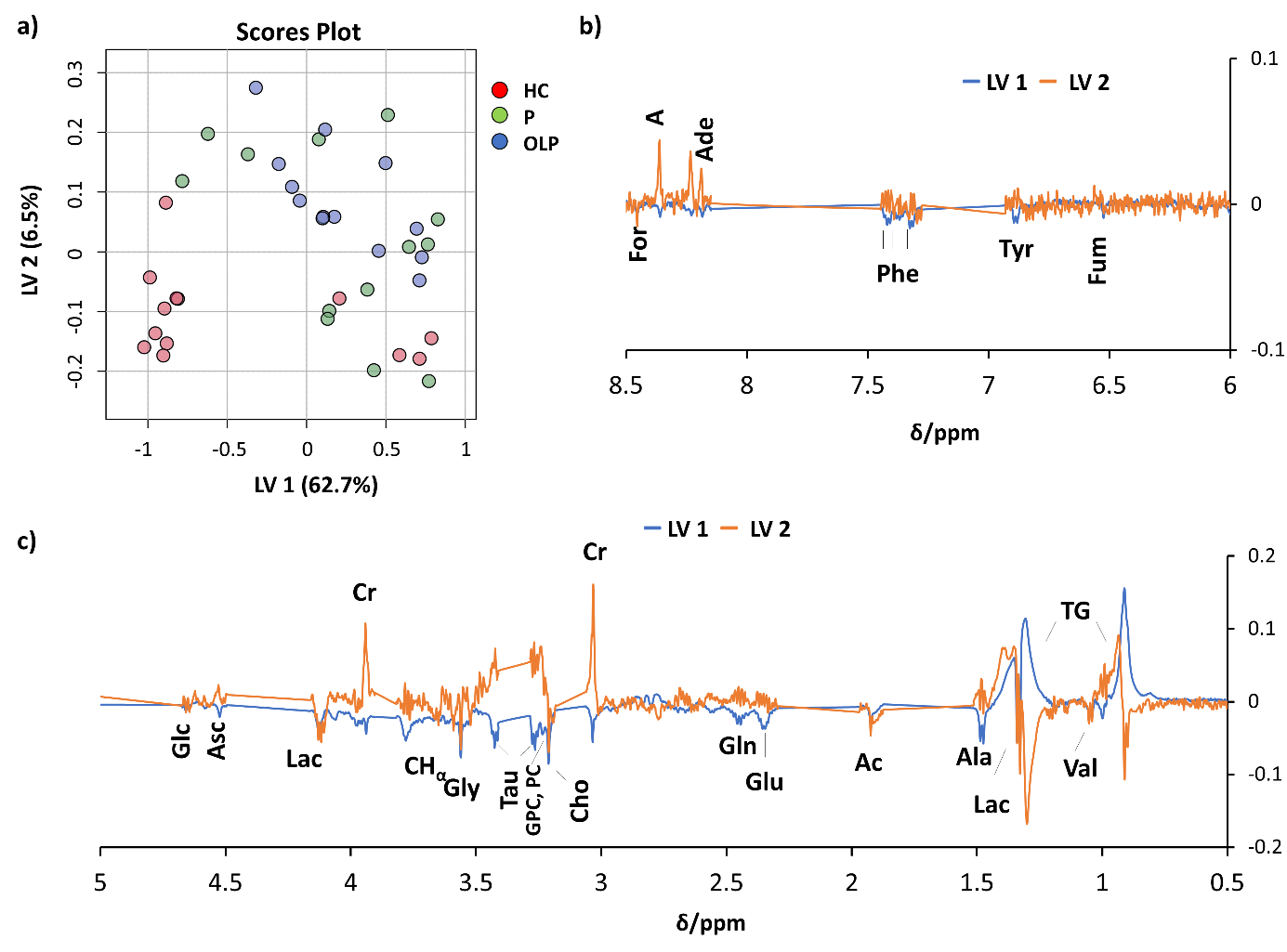


**Figure S1.** PLS-DA scores plot (a) and LV1 and LV2 loadings (aromatic region, b, aliphatic region, c) obtained from ^1^H HR-MAS NMR CPMG spectra of HC (red), P (green), and OLP (blue) tissue samples. For: formate, A: adenosine, Ade: adenine, Phe: phenylalanine, Tyr: tyrosine, Fum: fumarate, Glc: glucose, Asc: ascorbate, Lac: lactate, Cr: creatine, CHα: signals of CHα from alanine, glutamine and glutamate, Gly: glycine, Tau: taurine, GPC: glycerophosphocholine, PC: phosphocholine, Cho: choline, Gln: glutamine, Glu: glutamate, Ac: acetate, Ala: alanine, Val: valine, TG: triglyceride.


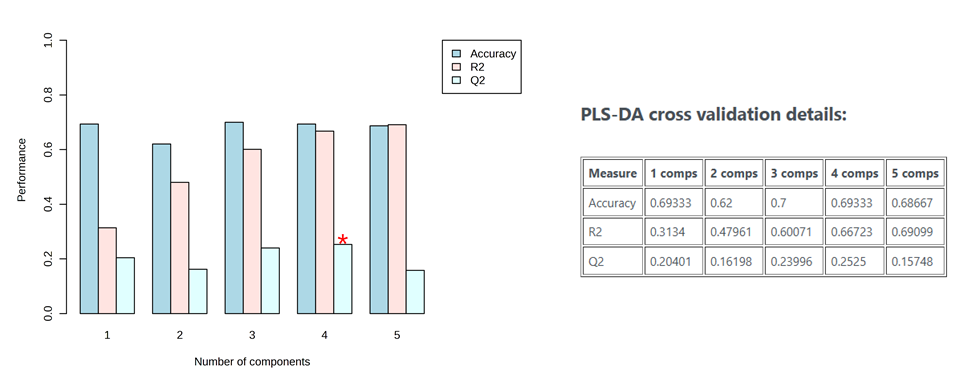


**Figure S2**. PLS-DA cross-validation details from deconvoluted HR-MAS NMR data obtained from tissues (Figure 3).


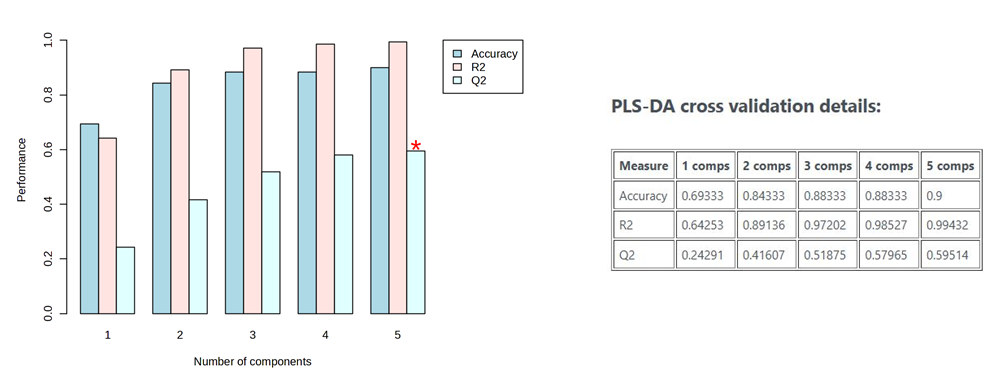


**Figure S3**. PLS-DA cross-validation details from saliva NMR data (Figure 4).
